# Validation of a monoclonal antibody-based immunofluorescent assay to detect *Burkholderia pseudomallei* in blood cultures

**DOI:** 10.1093/trstmh/trw079

**Published:** 2017-02-01

**Authors:** Adul Dulsuk, Suporn Paksanont, Adisak Sangchankoom, Peeraya Ekchariyawat, Rungnapa Phunpang, Yaowaruk Jutrakul, Narisara Chantratita, T. Eoin West

**Affiliations:** a Mahidol-Oxford Tropical Medicine Research Unit, Faculty of Tropical Medicine, Mahidol University, Bangkok, 10400, Thailand; b Department of Microbiology and Immunology, Faculty of Tropical Medicine, Mahidol University, Bangkok, 10400, Thailand; c Department of Medical Technology, Udon Thani Regional Hospital, Udon Thani, 41000, Thailand; d Division of Pulmonary and Critical Care Medicine, Department of Medicine, University of Washington, Seattle, WA, 98104-2499, USA; e International Respiratory and Severe Illness Center, University of Washington, Seattle, WA, 98104, USA

**Keywords:** Bacteremia, *Burkholderia pseudomallei*, Diagnosis, Immunofluorescent assay, Melioidosis, Thailand

## Abstract

**Background:**

Identification of *Burkholderia pseudomallei*, the cause of melioidosis, using routine methods takes several days. Use of a monoclonal antibody-based immunofluorescent assay (IFA) on positive blood cultures may speed diagnosis.

**Methods:**

We tested the diagnostic accuracy of the IFA on 545 blood cultures positive for Gram-negative organisms at Udon Thani Hospital, Thailand, between June 2015 and August 2016.

**Results:**

Sensitivity of the IFA was 100% and specificity was 99.6%. The median decrease in time to pathogen identification between the IFA result and routine methods was 28 h (IQR 25–51), p<0.0001.

**Conclusions:**

The IFA accurately expedites the diagnosis of melioidosis.

## Introduction

Melioidosis is caused by infection with the Gram-negative bacillus *Burkholderia pseudomallei*. Northeast Thailand is a hyperendemic zone for melioidosis due to the widespread presence of *B. pseudomallei* in the soil and water in this region.^1,2^ The clinical presentation of melioidosis is difficult to distinguish from other aetiologies of blood stream infection, pneumonia and sepsis. Due to intrinsic antibiotic resistance of *B. pseudomallei*, treatment of melioidosis requires the third generation cephalosporin ceftazidime or a carbapenem for at least 2 weeks followed by up to 20 weeks of trimethoprim-sulfamethoxazole. Mortality rates from melioidosis in Northeast Thailand average 40%.^3^ Rapid diagnosis is therefore essential. However, definitive diagnosis of melioidosis requires culture and subsequent identification of the organism which may take several days.

Previous studies have evaluated diagnostic strategies to accelerate the identification of *B. pseudomallei* and to ensure initiation of appropriate antibiotic treatment. One such strategy is the use of a monoclonal antibody-based immunofluorescent assay (IFA) to test blood cultures that flag positive. The IFA monoclonal antibody (mAb) is 4B11, which is specific for an exopolysaccharide of *B. pseudomallei*.^4,5^ This assay has been successfully used at Sappasithiprasong Hospital, Ubon Ratchathani, Thailand.^5^ We report here on the successful implementation of the IFA assay at another referral hospital in a hyperendemic melioidosis zone, Udon Thani Hospital, Udon Thani, Thailand.

## Materials and methods

We implemented the IFA on blood culture specimens collected by hospital staff from June 2015 to August 2016. This was done as part of a large prospective multi-center study of melioidosis. Routine practice is to take 10 ml of blood for adults or 5 ml of blood for children and inoculate this into a 30 ml bottle (Aerobic BacT/Alert FA Plus; Biomérieux, Marcy-l′ Étoile, France). The bottle is incubated in an automated BacT/ALERT 3D instrument at 37°C. Once the instrument flags a bottle as positive, it is removed for Gram stain and sub-culture. Bacterial isolates are subsequently identified using standard laboratory methodology derived from the Work Manual for Laboratory Identification of Bacteria and Fungi for Regional and General Hospitals, Department of Medical Science, Ministry of Public Health, Thailand. The IFA was performed once daily on 1 ml of fluid taken from blood culture bottles identified as positive by the BacT/ALERT 3D instrument and determined to have Gram-negative bacteria by Gram stain. In a Class II biosafety cabinet, 10 µl of working mAb-IFA reagent containing 5 µg/ml of mAb 4B11 and 20 µg/ml of Alexa Fluor 488 conjugated-goat anti-mouse IgG (Molecular Probes, Carlsbad, CA, USA) in phosphate-buffered saline (PBS).^4,5^ were mixed with an equal volume of blood culture fluid on a glass slide, a coverslip applied, and the slide left at room temperature for 10 min. Bacteria were observed using a fluorescent microscope (Olympus, Tokyo, Japan) at 1000x magnification.

## Results

A total of 553 sequential blood cultures were positive with Gram-negative bacilli during the study period. IFA was performed on 545 specimens. The IFA results for these specimens were compared to standard techniques used to identify *B. pseudomallei* (Table 1). The sensitivity of IFA was 100%, specificity 99.6%, positive predictive value 97.4%, and negative predictive value 100%. The two discordant specimens that were positive by IFA but negative for *B. pseudomallei* by routine methods were identified as *Escherichia coli*. One of these *E. coli* isolates cultured on agar was available for subsequent testing with IFA and the result was negative.

**Table 1.** Diagnostic accuracy of an immunofluorescent assay for *Burkholderia pseudomallei*

IFA	Routine identification methods		Total
	Positive	Negative	
Positive	74	2	76
Negative	0	469	469
Total	74	471	545

IFA: immunofluorescent assay.

For 533 specimens with reliable timestamp data available, the median time from collection of the blood culture specimen to identification of *B. pseudomallei* using IFA was 41 h (IQR 24–47). The median time from collection of the blood culture specimen to identification of *B. pseudomallei* using standard techniques was 72 h (IQR 65–94). The median decrease in time between the IFA result and standard technique result was 28 h (IQR 25–51), p<0.0001.

## Discussion

Our results confirm the excellent test characteristics of the monoclonal antibody IFA for *B. pseudomallei* identification in positive blood cultures in a melioidosis-endemic area. These characteristics are comparable to the original report of this test elsewhere in Thailand,^5^ confirming validity of the assay. Notably, in the previous study the IFA was performed on all positive blood cultures, whereas in this study we restricted testing to positive blood cultures with Gram-negative organisms. The reason for false positive results for *E. coli* isolates does not appear to be due to cross-reactivity of the monoclonal antibody to *E. coli* but may be due to other unknown factors including operator error.

We also provide precise quantitation of the decreased time to bacterial identification with the IFA. We show that the diagnosis of melioidosis can be made at least one day earlier than with standard diagnostic methods, a statistically and clinically significant decrease. In light of the high mortality rate of hospitalized patients with melioidosis in Thailand, this result is highly informative for clinicians and facilitates administration of appropriate therapy for *B. pseudomallei.*

A limitation to this study is that one trained technician as part of a research protocol performed the IFA. The diagnostic accuracy of the test and timeliness of the results may be lower when implemented in a busy clinical microbiology laboratory. However, the IFA can typically be performed in less than 30 min. Chemical inactivation of the bacteria prior to IFA testing is presently being investigated to increase safety for laboratory staff.

### Conclusions

We show excellent diagnostic accuracy of an IFA for detection of *B. pseudomallei* in blood cultures at a Thai referral hospital, and demonstrate a reduced time to diagnosis. These data provide important confirmation of validity of the IFA and support the widespread use of the test for prompt diagnosis of melioidosis in areas where the infection is endemic.
